# Complexation of C6-Ceramide with Cholesteryl Phosphocholine – A Potent Solvent-Free Ceramide Delivery Formulation for Cells in Culture

**DOI:** 10.1371/journal.pone.0061290

**Published:** 2013-04-19

**Authors:** Pramod Sukumaran, Max Lönnfors, Otto Långvik, Ilari Pulli, Kid Törnquist, J. Peter Slotte

**Affiliations:** 1 Cell Biology, Åbo Akademi University, Turku, Finland; 2 Biochemistry, Department of Biosciences, Åbo Akademi University, Turku, Finland; 3 Laboratory of Organic Chemistry, Department of Natural Sciences, Åbo Akademi University, Turku, Finland; 4 Minerva Foundation Institute of Medical Research, Biomedicum Helsinki, Helsinki, Finland; MUSC SC College of Pharmacy, United States of America

## Abstract

Ceramides are potent bioactive molecules in cells. However, they are very hydrophobic molecules, and difficult to deliver efficiently to cells. We have made fluid bilayers from a short-chain D-*erythro*-ceramide (C6-Cer) and cholesteryl phosphocholine (CholPC), and have used this as a formulation to deliver ceramide to cells. C6-Cer complexed with CholPC led to much larger biological effects in cultured cells (rat thyroid FRTL-5 and human HeLa cells in culture) compared to C6-Cer dissolved in dimethyl sulfoxide (DMSO). Inhibition of cell proliferation and induction of apoptosis was significantly more efficient by C6-Cer/CholPC compared to C6-Cer dissolved in DMSO. C6-Cer/CholPC also permeated cell membranes and caused mitochondrial Ca^2+^ influx more efficiently than C6-Cer in DMSO. Even though CholPC was taken up by cells to some extent (from C6-Cer/CholPC bilayers), and was partially hydrolyzed to free cholesterol (about 9%), none of the antiproliferative effects were due to CholPC or excess cholesterol. The ceramide effect was not limited to D-*erythro*-C6-Cer, since L-*erythro*-C6-Cer and D-*erythro*-C6-dihydroCer also inhibited cell priolifereation and affected Ca^2+^ homeostasis. We conclude that C6-Cer complexed to CholPC increased the bioavailability of the short-chain ceramide for cells, and potentiated its effects in comparison to solvent-dissolved C6-Cer. This new ceramide formulation appears to be superior to previous solvent delivery approaches, and may even be useful with longer-chain ceramides.

## Introduction

Sphingolipids constitute an important group of lipids with specific structural and functional properties [1]–[3]. Contrary to glycerolipids, in which glycerol is used to link up to three functional groups to create more complex lipids, sphingolipid structure is based on a long chain base, to which additional functional groups can be attached [4]. During long chain base synthesis, sphinganine is formed [5]. After acylation of its NH_2_ group, the resulting dihydroceramide can be used for synthesis of more complex sphingolipids. However, very often a desaturase will introduce a *trans* double bond between carbons 4 and 5 in the long chain base, thus forming ceramide [6]. Further modifications of ceramide may yield ceramide-1-phosphate, sphingomyelin, cerebrosides, and gangliosides [7]. Degradation of ceramide with ceramidases gives rise to sphingosine and free fatty acid [8], of which the sphingosine may be phosphorylated [9]. While the sphingomyelins and glycosphingolipids have important structural function in cell membranes [10]–[12], ceramide and the phosphorylated forms of both ceramide and sphingosine are potent signaling lipids in cells [7], [9], [13]–[15]. However, the more complex sphingolipids serve as precursors in the intricately regulated generation of bioactive sphingolipid intermediates. Because functional modifications of ceramides generate structural as well as bioactive molecules, ceramide metabolism is considered to be the hub in sphingolipid turnover [5].

The bioactivity of ceramide has been showed to be important for the regulation of cell growth, proliferation and differentiation [16]–[18], and for cell senescence, necrosis and apoptosis [19]–[21]. Some of ceramide’s effects have been shown to involve activation of different protein phosphatases [22]. However, many other action mechanisms are likely. For instance, ceramide down-regulates the HERG potassium channel [23], and affect the resting membrane potential of thyroid FRTL-cells by processes involving protein kinase C zeta [24]. Ceramides have also been suggested to form pores in bilayer membranes, and more recently also in mitochondrial outer membranes, thus possibly facilitating apoptosis [25]–[27]. Since so many different ceramide species exists in cells, and because ceramide generation can be highly compartmentalized, it is likely that different signaling pathways are influenced differently, increasing the challenge of understanding the various roles ceramide can have in cell signaling events.

Ceramide is a very hydrophobic molecule, and has extremely poor aqueous solubility. This property has greatly hampered studies on the biological activity of ceramides. While ceramides can be generated in cells by activation of endogenous sphingomyelinases [28], [29], or by using bacterial sphingomyelinases, direct addition of natural ceramides to cells has been difficult. This is apparently the main reason why many scientist have used short-chain ceramide analogs (dissolved in organic solvent) to study ceramide effects in cells, since their efficient delivery to cells has been possible. Ceramides do not appear to form similar types of water-soluble complexes with cyclodextrins, as cholesterol does, although in one early study the use of alpha-cyclodextrin facilitated ceramide synthesis, and may have formed some complexes with ceramide [30]. However, liposomal complexes of ceramides and phospholipids can be used instead of solvent to deliver ceramides to cells. As expected, shorter-chain analogs were more potent in affecting cell viability when compared to more physiological ceramides [31], [32].

**Figure 1:**
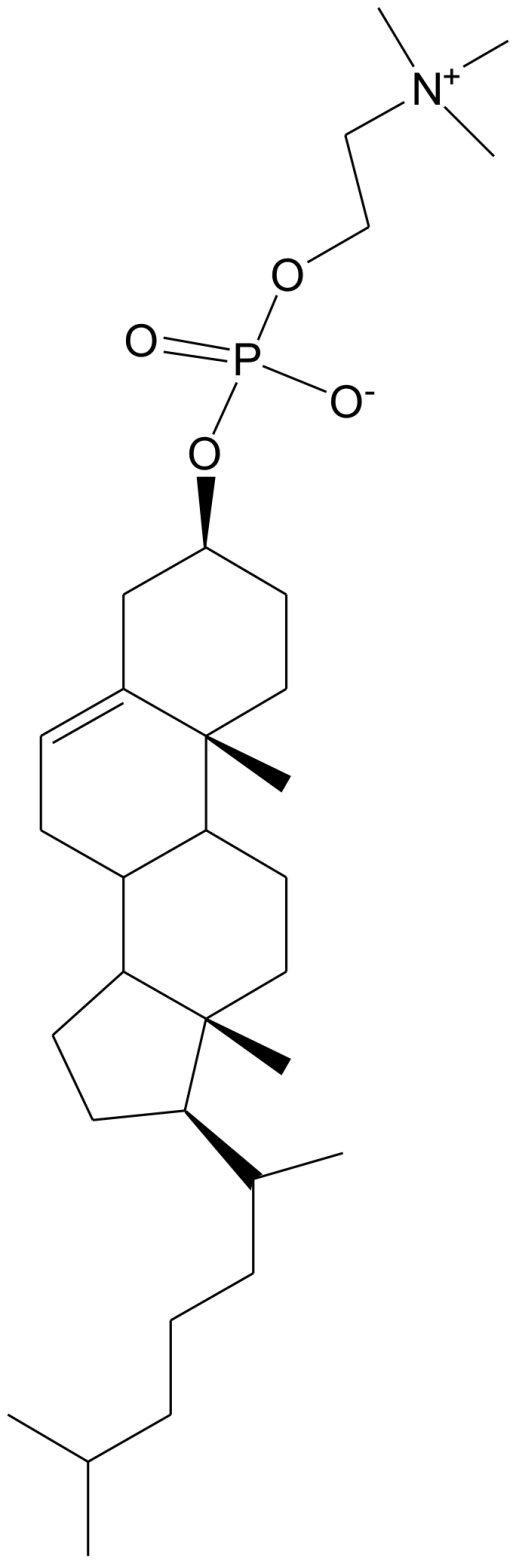
Chemical structure of CholPC.

We have recently shown that ceramides can form fluid bilayers with cholesteryl phosphorylcholine (CholPC – see Figure 1 for structure) [33]. The fluid nature of the bilayers was evident even when palmitoyl ceramide was used with CholPC. Apparently the CholPC/ceramide formulation is stable, since the phosphocholine head group attached to cholesterol can protect both molecules from unfavorable interactions with water. Similar bilayers were previously shown to be formed by CholPC and dimyristoyl glycerol [34]. We have in this study compared the bioactivity of *N*-hexanoyl ceramide (C6-Cer) in two cell types (HeLa and rat thyroid FRTL cells) when presented to cells either as a complex with CholPC or dissolved in DMSO. Our results show that CholPC-complexed C6-Cer was much more potent in causing inhibition of proliferation, apoptosis, and disturbed Ca^2+^ homeostasis, as compared to solvent-delivered C6-Cer. We suggest that C6-Cer in a bilayer with CholPC provides a much better bioavailability, and can be successfully used to deliver ceramides to cells in culture.

## Results

### Cellular Uptake of [^3^H]C6-Cer from Different Formulations

**Figure 2:**
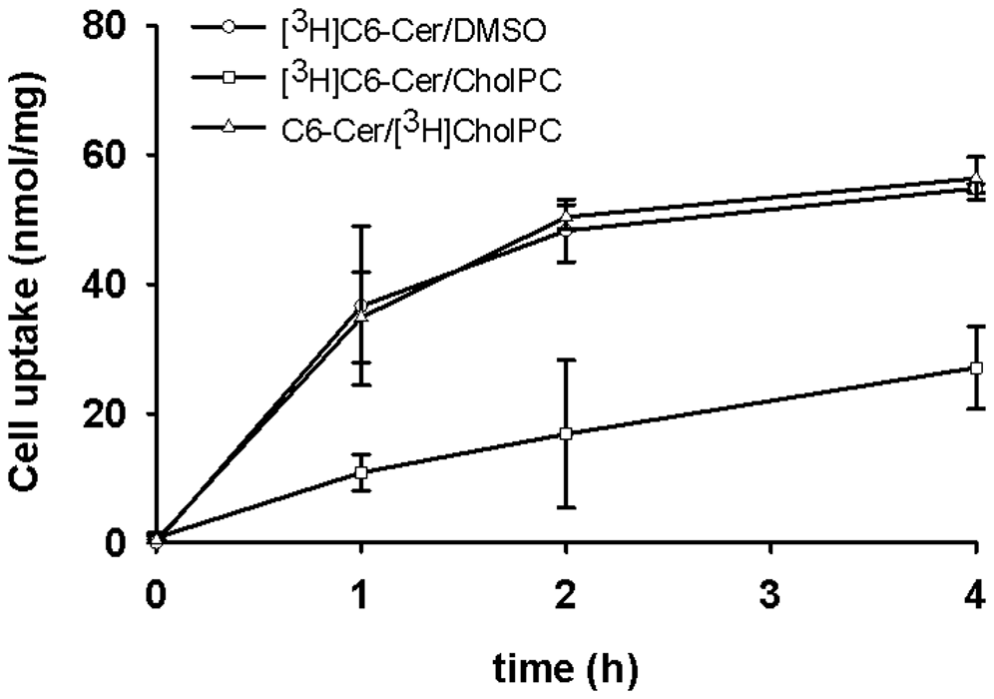
Cellular incorporation of [^3^H]C6-Cer or [^3^H]CholPC. FRTL-5 cells were exposed for the indicated time to 0.05 mM of either [^3^H]C6-Cer/CholPC, [^3^H]C6-Cer/DMSO, or C6-Cer/[^3^H]CholPC. The uptake of lipids was normalized to cell protein, and each value is an average from 6 dishes±SEM. The zero time labeling was about 1300 cpm/dish (with each label), and was substracted from later time point values.

To measure the uptake and cellular association of [^3^H]C6-Cer, FRTL-5 cells were exposed to 0.05 mM of [^3^H]C6-Cer (∼0.011 µCi/mmol) complexed with CholPC or dissolved in DMSO. Alternatively, [^3^H]CholPC was used (0.05 mM, ∼0.057 µCi/mmol), complexed with C6-Cer, and its cellular uptake was followed. As shown in Figure 2, cell-associated label (both [^3^H]CholPC and [^3^H]C6-Cer) increased curvi-linearly with time (0.4 h). Comparing the cellular uptake of [^3^H]C6-Cer from either [^3^H]C6-Cer/CholPC or [^3^H]C6-Cer/DMSO, it was observed that cell association of [^3^H]C6-Cer appeared to be more efficient from DMSO solvent compared to CholPC bilayers. Exposure of cells to C6-Cer/[^3^H]CholPC vesicles led to a time-dependent incorporation of [^3^H]CholPC into the cells, the extent of which was larger than seen with C6-[^3^H]Cer uptake.

Cancerous cells in culture have been shown to convert C6-Cer rapidly to C6-SM or C6-glycosylceramide, the actual conversion route being subject to the initial C5-Cer concentration cells were exposed to [42]. We have not further examined the possible conversion of C6-Cer into other sphingolipid classes in the FRTL-5 cells. However, since CholPC metabolism in cells has not been previously addressed, we deemed it important to examine the cellular fate of internalized [^3^H]CholPC. We examined the extent of hydrolysis (to [^3^H]cholesterol) and the distribution of label into [^3^H]cholesteryl esters, the latter being indicative of cholesterol mass influx into the cells. After a 4 h exposure of cells to C6-cer/[^3^H]CholPC (0.05 mM of each component), lipid analysis revealed than 8.9±1.9% of [^3^H]CholPC had been hydrolyzed to [^3^H]cholesterol. Only a small fraction of the label was found as [^3^H]cholesteryl esters (0.5±0.1). The low labeling of cholesteryl esters indicate that the increase in cellular free cholesterol mass was not significant, since excess of free cholesterol is known to rapidly stimulate cholesteryl ester synthesis [43].

### Ceramide Inhibits the Proliferation of FRTL-5 and HeLa Cells

**Figure 3:**
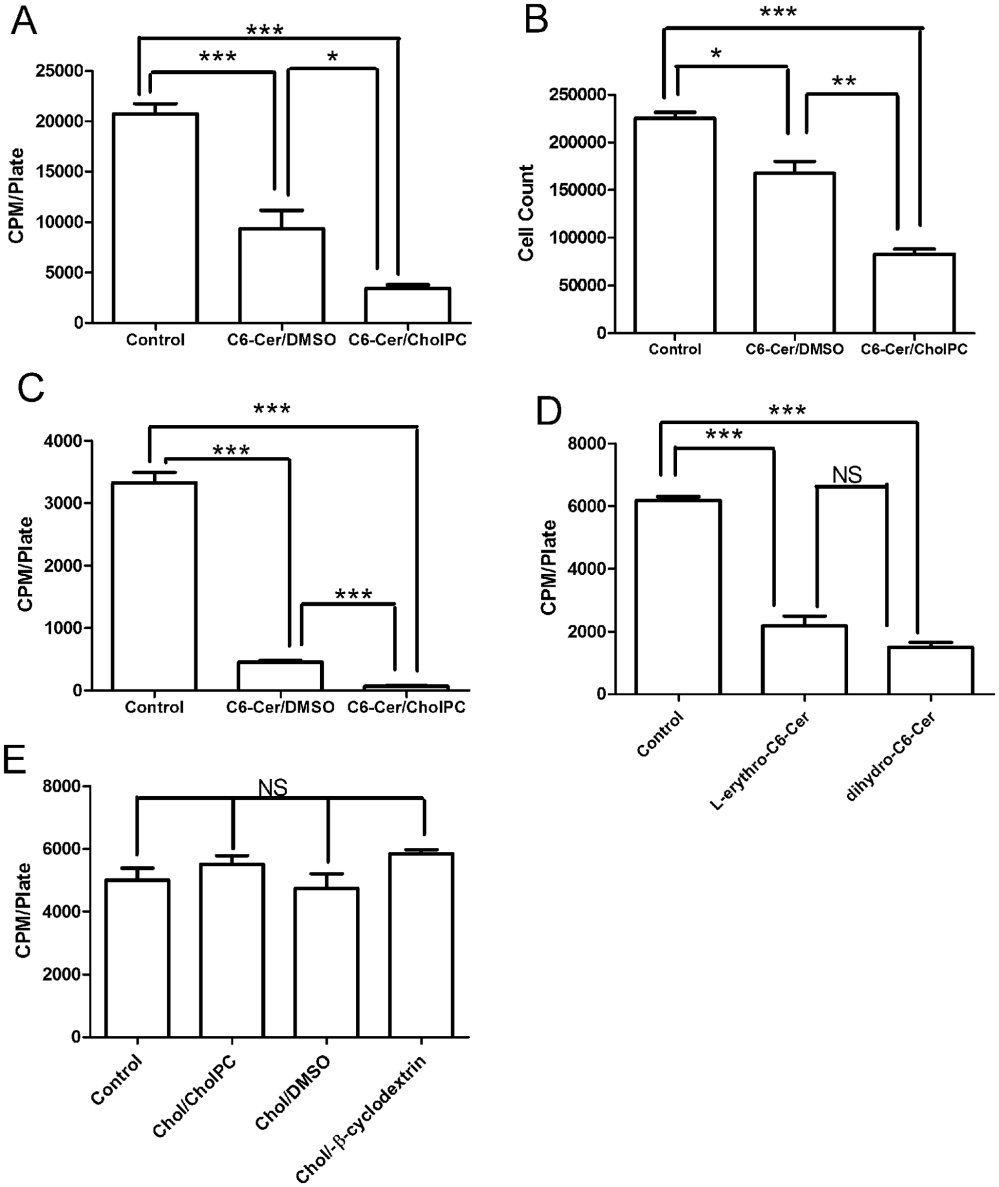
Effect of C6-Cer on cell proliferation. A. FRTL-5 cells were preincubated for 48 h with 0.05 mM C6-Cer/CholPC or C6-Cer/DMSO. [^3^H]Thymidine incorporation into cellular DNA during the last 4 h was determined. B. Cell proliferation measurement using cell count. Effect of C6-Cer on cell proliferation on FRTL-5 was measured by counting the cells after preincubation for 48 h with 0.05 mM C6-Cer/CholPC or C6-Cer/DMSO. DMSO alone was used as control. Each value gives the amount of cells per plate. C. HeLa cells were incubated with 0.05 mM C6-Cer/CholPC or C6-Cer/DMSO for 12 h. [^3^H]Thymidine incorporation into cellular DNA during the last 4 h was determined. D. FRTL-5 cells were exposed to 0.05 mM L-erythro-C6-Cer/CholPC or C6-dihydroCer/CholPC for 24 h after which [^3^H]thymidine incorporation into cellular DNA during the last 4 h was determined. E. FRTL-5 cells were exposed for 48 h to 0.05 mM Chol/CholPC, Chol/DMSO, or Chol/m-β-cyclodextrin after which [^3^H]thymidine incorporation into cellular DNA during the last 4 h was determined. Each value is the mean ± SEM of at least 3 independent experiments. *P<0.05; **P<0.01; ***P<0.001.

The proliferation of FRTL-5 cells has been shown to be inhibited by ceramide [44], [45]. We therefore compared the effects of the different C6-Cer formulations on the rate of [^3^H]thymidine incorporation into cellular DNA (Fig. 3A). FRTL-5 cells exposed to 0.05 mM of C6-Cer/CholPC or C6-Cer dissolved in DMSO for 48 h had lower [^3^H]thymidine incorporation compared to control cells (DMSO exposure only), and the effect was much larger in cells exposed to C6-Cer/CholPC. The results were confirmed by counting the number of cells after 48 hrs of treatment (Fig. 3B). Similar data were obtained when HeLa cells were exposed for 12 h to 0.05 mM C6-Cer, either in complex with CholPC, or dissolved in DMSO (Fig. 3C). The antiproliferative effects of C6-Cer was also seen when FRTL-5 cells were exposed to 0.05 mM C6-ceramide made from L-erythro-sphingosine, or from D-erythro-sphinganine (Fig. 3D).

To exclude the possibility that CholPC was the cause of inhibiting cell growth, we measured the effect of Chol/CholPC, Chol/m-β-cyclodextrin, or Chol/DMSO formulations on FRTL-5 proliferation (0.05 mM CholPC or Chol). As seen in Figure 3E, exposure of FRTL-5 cells to any of the C6-Cer-free Chol or CholPC formulations showed no significant effects on [^3^H]thymidine incorporation, indicating no effect (inhibitory or stimulatory) on FRTL-5 proliferation.

### Effect of C6-Cer on Cytosolic Ca^2+^ Homeostasis in HeLa Cells

**Figure 4:**
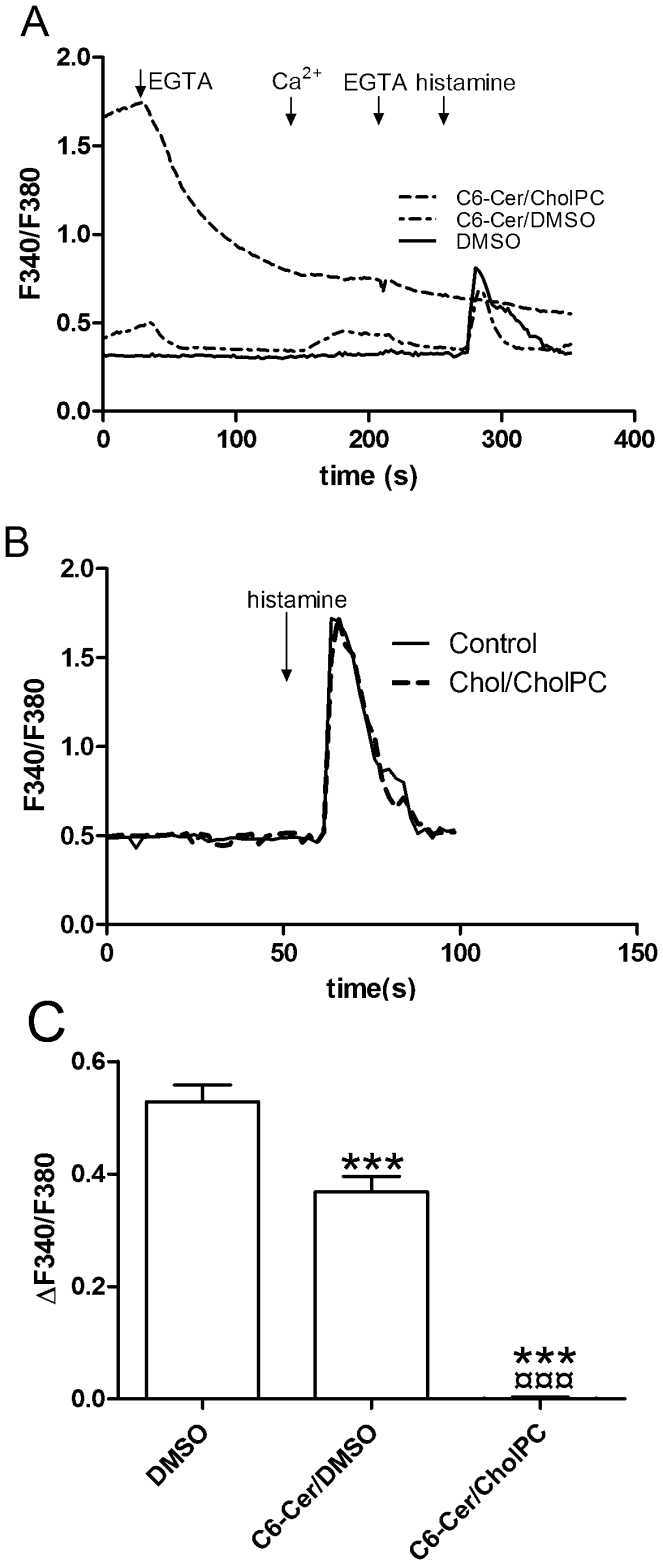
C6-Cer/CholPC disrupts cytosolic calcium homeostasis in HeLa cells. The cells (panel A) were preincubated for 90 min with 0.05 mM C6-Cer/CholPC or C6-Cer/DMSO, and changes in intracellular Ca^2+^ levels were measured using Fura 2-AM. DMSO alone was used as control. The cells were stimulated as indicated by the arrows (150 µM EGTA, 1 mM Ca^2+^, and 10 µM histamine). The Ca^2+^ traces are averages from 33 individually measured cells that were randomly selected. In panel B, cells were instead treated with Chol/CholPC or DMSO (control) and stimulated with 10 µM histamine in calcium containing HBSS buffer. The Ca^2+^ traces are averages from 34 individually measured cells that were randomly selected. Panel C shows the quantification of the change in F340/F380 from basal to maximal values upon 10 µM histamine stimulation in panel A. The data were analyzed using one-way Anova and Bonferroni’s multiple comparison test (***p<0.001 compared with DMSO; ¤¤¤p<0.001 compared with C6-Cer).

Ceramide has been shown to greatly affect intracellular Ca^2+^ homeostasis in FRTL-5 cells [46]. To investigate the effect of C6-Cer on cytosolic free Ca^2+^ concentrations ([Ca^2+^]_cyt_), the Ca^2+^ indicator Fura 2-AM was employed. As the HeLa cells appeared to be much more sensitive to the different ceramid formulations, we used HeLa cells in these assays. Prior to Ca^2+^ measurements, the cells were preincubated with C6-Cer/CholPC or C6-Cer/DMSO (0.05 mM) for 90 min, respectively. Then cells were treated as indicated in Figure 4A. After a 90-min preincubation of HeLa cells with C6-Cer/CholPC, the cells showed markedly increased basal [Ca^2+^]_cyt_, whereas preincubation of cells with C6-Cer/DMSO did not significantly increase basal [Ca^2+^]_cyt_. The initial basal Fura 2-AM fluorescence ratios (F340/F380) were 1,881±0,098 for C6-Cer/CholPC treated cells, 0,5137±0,048 for C6-Cer/DMSO and 0,3315±0,008 for DMSO, respectively. Also, C6-Cer/CholPC treated cells showed a clear and acute reduction of [Ca^2+^]_cyt_ in response to addition of EGTA (final concentration 150 µM), indicating high Ca^2+^ permeability of the plasma membrane (Fig. 4A). This was not seen with C6-Cer/DMSO or DMSO alone. Furthermore, C6-Cer/CholPC treatment inhibited the cytosolic Ca^2+^ response upon stimulation of the IP_3_ sensitive Ca^2+^ stores with histamine (final concentration 10 µM; Fig. 4A and Fig. 4C). Exposure of cells to the Chol/CholPC formulation gave no effect on cytosolic Ca^2+^ levels (Fig. 4B). These results show that C6-Cer/CholPC potently disrupts the handling of [Ca^2+^]_cyt_ in response to changes in extracellular [Ca^2+^], as well as when the cells are challenged with the IP_3_ generating agonist histamine.

### Mitochondrial Ca^2+^ Uptake is Reduced after C6-Cer/CholPC, C6-Cer/DMSO, and Dihydro-C6-Cer/CholPC Treatment

**Figure 5:**
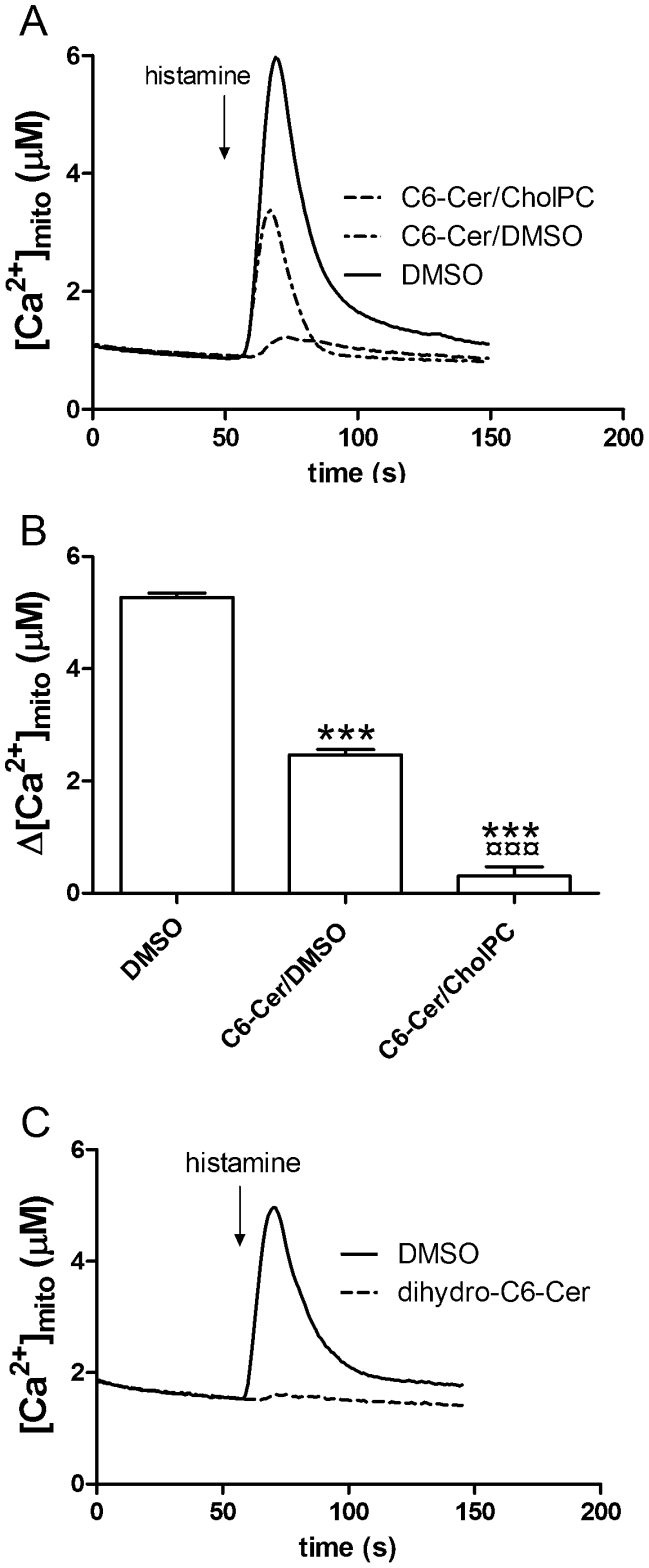
C6-Cer/CholPC and C6-dihydroCer reduce mitochondrial calcium uptake. HeLa cells were preincubated for 180 min with 0.05 mM C6-Cer/CholPC or C6-Cer/DMSO (panels A and B), and changes in intracellular Ca^2+^ levels were measured using mtAEQ. DMSO was added to control cells. Panel A shows kinetics of changes in mitochondrial Ca^2+^ after challenge with histamine. In panel B, the change in Ca^2+^ response was quantitated. The cells were challenged with 100 µM histamine as indicated by the arrow. Panel C shows kinetics of the Ca^2+^ response after180 min exposure of cells to C6-dihydroCer. Traces (panel A and C) are averages of 3 measurements, each representing the average luminescence from a cell population of 150 000–200 000 cells. In panel B, the bar shows the average change in [Ca^2+^]_mito_ during histamine-induced Ca^2+^ release (± SEM, n = 3). The data were analyzed using one-way Anova and Bonferroni’s multiple comparison test (***p<0.001 compared with DMSO; ¤¤¤p<0.001 compared with C6-Cer).

Ceramides have been shown to induce channel formation in both bilayer membranes and in mitochondrial outer membranes, possibly explaining the ceramide-induced increase in the permeability of mitochondrial outer membranes [26], [27]. Furthermore, as the histamine-evoked release of ER calcium was severely hampered, and as the ER and mitochondria are in close contact with each other [47], we therefore wanted to investigate the effects of the ceramide formulations on mitochondrial Ca^2+^ handling. For the measurements of mitochondrial Ca^2+^ concentrations ([Ca^2+^]_mito_), we used mitochondrially targeted recombinant aequorin. The cells were preincubated for 180 min with C6-Cer/CholPC, C6-Cer/DMSO or dihydro-C6-Cer/CholPC (0.05 mM), and stimulated with 100 µM histamine. Mitochondrial Ca^2+^ uptake was nearly abolished in C6-Cer/CholPC treated cells and reduced in C6-Cer/DMSO treated cells (Fig. 5A&B). Also, the cells treated with dihydro-C6-Cer/CholPC showed an almost complete inhibition of mitochondrial calcium uptake (Fig. 5C).

### Ceramide Induces Apoptosis in the FRTL-5 Cells

**Figure 6:**
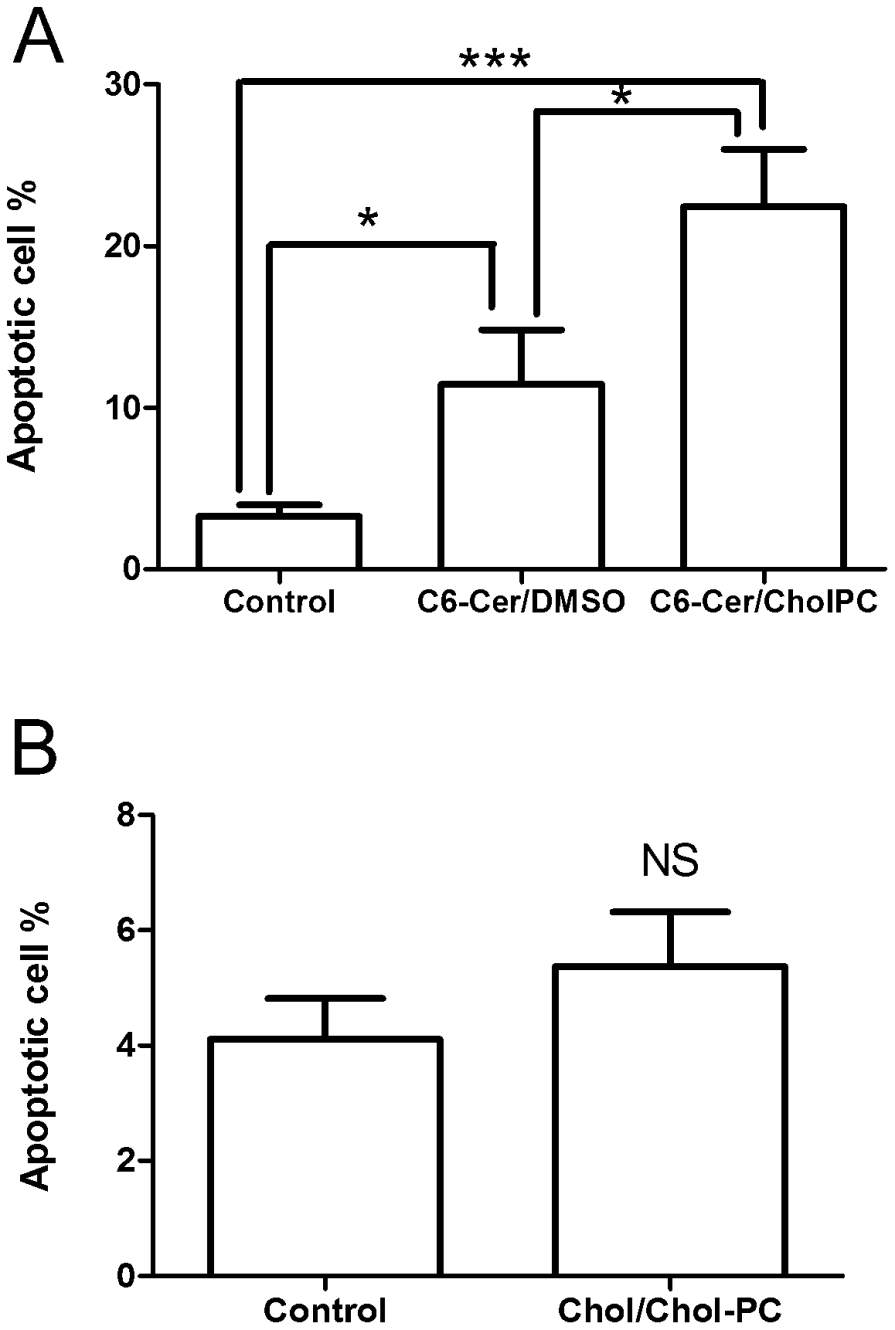
Induction of apoptosis in FRTL-5 cells by C6-Cer. A. The cells were exposed for 48 hrs to C6-Cer/CholPC or C6-Cer/DMSO (0.05 mM), and the fraction of apoptotic cells was measured. Each bar value is the mean±SEM of 3 different experiments. *p<0.05, ***p<0.001. B. Induction of apoptosis in FRTL-5 cells by Chol/Chol-PC. The cells were exposed for 48 hrs to Chol/Chol-PC (0.05 mM), and the fraction of apoptotic cells was measured. Each bar value is the mean±SEM of 3 different experiments (NS = no significance).

Previous studies have shown that ceramide induces apoptosis in FRTL-5 cells [45]. To test the effects on apoptosis of the different C6-Cer formulations, FRTL-5 cells were exposed to C6-Cer/CholpC or C6-Cer/DMSO (0.05 mM) for 48 h. The proportion of apoptotic cells in controls and C6-Cer exposed cells was measured with FACS, and results calculated using Flowing Software v 2.5 (Fig. 6). Less than 5% of the cells were apoptotic when not exposed to C6-Cer, whereas the fraction of apoptotic cells increased markedly in C6-Cer treated cells (Fig. 6A). C6-Cer/CholPC was significantly more effective in inducing apoptosis in the FRTL-5 cells compared to the C6-Cer/DMSO formulation. As a control experiment the apoptotic cell percentage was also calculated for the cells treated with Chol/Chol-PC for 48 h (Fig. 6B).

## Discussion

Ceramides are potent bioactive molecules, known to affect many cellular processes, by directly modifying enzyme activities [22], [48]–[50], or possibly by creating ceramide-enriched ordered domains [51], [52], and even membrane pores allowing for membrane permeabilization [25], [53]. The latter mechanism may be relevant for ceramide-induced cytochrome leakage from mitochondria, an early event in the apoptotic pathway [54]. For studies of the effects of ceramides on cellular processes, ceramides must either be generated (e.g., sphingomyelinase activation), or transferred from extracellular vehicles to cells. The latter approach is hampered by the hydrophobicity of ceramides, which prevent efficient and spontaneous transfer through the aqueous phase.

In a previous study, we showed that ceramides (e.g., 16∶0-Cer) can form fluid bilayers with CholPC [33]. In the present study, we have shown that C6-Cer complexed with CholPC into bilayers have a much higher potency to inhibit cell growth and induce apoptosis, when compared to C6-Cer dissolved in DMSO. Using [^3^H]sphingosine-labeled C6-Cer, we observed that short-term (up to 4 h, Fig. 2) cellular association of C6-Cer was higher from DMSO than from CholPC bilayers. However, since the C6-Cer/CholPC formulation was superior to the DMSO formulation in all cellular responses to C6-Cer, we believe that C6-Cer addition to cells from DMSO led to crystallization of the C6-Cer when diluted to the cell growth medium. Such crystals may stick to cells more than C6-Cer/CholPC vesicles, and hence give rise to higher association of the [^3^H]label with cells.

The anti-proliferative effect of short-chain ceramides is well documented [16], [18]. We observed that C6-Cer was very efficient in reducing cell proliferation, both in FRTL-5 and HeLa cells. The effect was significantly larger with C6-Cer/CholPC at 48 h compared to DSMO delivered C6-Cer (FRTL-5 cells, Fig. 2A). HeLa cells appeared to be even more sensitive to the antiproliferative effects of C6-Cer, irrespective of the delivery form (Fig. 3B). The antiproliferative effect of C6-Cer was also seen when the long-chain base had the unnatural L-erythro configuration [55], or when sphinganine (yielding dihydroceramide) was the long-chain base (Fig. 3C). Many ceramide effects are considered to be specific for the natural D-*erythro N*-acyl sphingosine species, and dihydroceramide has been shown to be inactive for many processes that ceramides influence [56]–[58]. Clearly, for cell growth inhibition, the D-erythro-sphingosine requirement was not absolute. However, our present data cannot distinguish between direct and indirect effects of ceramide causing the anti-proliferative effects. We also cannot exlude the possibility that some of the observed effects may arise from effects of C6-Cer on membrane protein and lipid redistribution.

CholPC by itself, although transferrable between C6-cer/CholPC vesicles and cell membranes (Fig. 2), and being partially hydrolyzed to free cholesterol, did not affect cell proliferation (FRTL-5 cells; Fig. 3B). Exposing cells to cholesterol dissolved either in DMSO or methyl-β-cyclodextrin also did not affect cell proliferation. Although cholesterol synthesis is important for normal progression of cell division and proliferation [59], excess cholesterol is considered toxic to cells. Enrichment of cholesterol in cells lead to increased phospholipid synthesis [38] and cholesteryl ester formation [60]. Since cellular cholesteryl ester formation was not markedly increased after exposure of FRTL-5 cells to CholPC-containing vesicles for 4 h, we conclude that cholesterol mass increase in the cells was not substantial after the treatment.

HeLa cells exposed to C6-Cer/CholPC did manifest an increased basal intracellular Ca^2+^ level, suggesting that the integrity of their plasma membranes may have been compromised by either C6-Cer or by CholPC. C6-Cer is the more likely the molecule causing the effect, since ceramides are known to affect Ca^2+^ levels in cells by several different mechanisms. In addition to making channels in the plasma membrane [53], [61], ceramides may deplete ER Ca^2+^ stores and evoke store-operated Ca^2+^ entry [62], or block store-operated Ca^2+^ entry [46]. Furthermore, by depolarizing the plasma membrane potential by blocking potassium channels [44], [61], and by collapsing the mitochondrial membrane potential [63], ceramides may also modulate cellular Ca^2+^ homeostasis. The dramatic increase in basal Ca^2+^ levels we observed in cells after incubation with C6-Cer/CholPC suggests that the ER was depleted. This depletion resulted in a substantial increase in basal cytosolic Ca^2+^ levels due to store-operated Ca^2+^ entry. The magnitude of the increase also suggests that C6-Cer/CholPC probably produced channels in the plasma membrane as well. The lack of Ca^2+^ uptake in the mitochondria after C6-Cer/CholPC probably was the result of the depleted ER Ca^2+^ store, as well as production of pores in the mitochondrial membranes [25]–[27]. Similar effects on cellular Ca^2+^ handling were also observed by C6-DMSO, although the magnitude was modest compared with that of C6-Cer/CholPC. Interestingly, C6-dihydroCer had the same effects on mitochondrial Ca2+ as C6-Cer, despite claims in the literature that C6-dihydroCer is unable to form ceramide pores in mitochondrial membranes [58].

Ceramides are widely known to cause apoptosis in cells. The mechanism(s) of action possibly include inhibition of survival pathways (pAkt inhibition [64], [65]), permeabilization of mitochondrial outer membranes [26], [27], [66], and caspase activation [67]. We observed dramatic increase in apoptosis in FRTL-5 cells exposed to C6-Cer (Fig. 6). Again the effect was significantly higher when C6-Cer was presented to cells in the form of CholPC-bilayers (compared to DMSO). Neither cholesterol nor Chol/CholPC formulations did affect the number of apoptotic cells. It is very likely that C6-Cer affects cells in different ways (possibly indirectly), leading to inhibition of cell proliferation, disturbed Ca^2+^ homeostasis, and apoptosis. What is clear from this study is that the means of ceramide delivery to cells dramatically affect the potency of the ceramide. Short-chain ceramides dissolved in DMSO are likely to precipitate when added to cell culture medium, and the bioavailability of “crystalline” ceramide is low. Formation of fluid bilayers which are enriched in C6-Cer (about 50 mol%) prevent crystallization of ceramide, and instead apparently allows for transfer of monomeric ceramide from the C6-cer/CholPC bilayers to cell membranes. However, we cannot rule out the possibility of limited fusion of C6-Cer/CholPC vesicles with cell membranes.

Long-chain ceramides also form fluid bilayers with CholPC, and these could possibly be used to deliver biologically relevant ceramide species to cells. However, the hydrophobicity (or acyl chain length) of complex lipids correlate well with their transfer efficiency between membrane structures [68], [69], and hence long-chain ceramides would be predicted to transfer more slowly than short-chain ceramides, even in formulations with CholPC. However, as shown in this study, the bioavailability of a short-chain ceramide complexed with CholPC was superior to DMSO-dissolved ceramide. It is difficult to directly compare the efficacy of short-chain ceramides complexed with CholPC to other formulations based on liposomal complexes [31], [32]. However, it is likely that ceramides would interact more preferably with phospholipids in liposomal compexes than with CholPC in our type of formulation. Such differences in co-lipid interactions could lead to significant differences in the bioavailablity of complexed ceramides. Clearly more studies are warranted to elucidate the benefits and disadvantages of ceramide/CholPC formulations as compared to ceramide/liposome formulations.

## Experimental

### Materials

Cholesterol, methyl-β-cyclodextrin, and dimethyl sulfoxide (DMSO) were obtained from Sigma/Aldrich (St. Louis, MO). D-*erythro*-sphingosine and hexanoic acid were purchased from Larodan (Malmö, Sweden). [1,2-^3^H(N)]Cholesterol (40–60 Ci/mmol) and [3-^3^H]D-erythro-sphingosine (15–30 Ci/mmol) were obtained from PerkinElmer (Waltham, MA, USA). Organic solvents were from Rathburn Chemicals Ltd (Walkerburn Scotland). Culture medium, serum, and hormones, except bovine TSH, were purchased from Invitrogen (Carlsbad, CA) and Sigma/Aldrich. Bovine TSH was obtained from Dr. A. F. Parlow and the National Hormone and Pituitary Program (National Institutes of Health, Bethesda, MD). Culture dishes were obtained from Falcon Plastics (Oxnard, CA). Fura 2-AM, penicillin/streptomycin, coelenterazine, and trypsin were from Invitrogen (Carlsbad, CA). Coon’s modified Ham F-12 and Dulbecco’s Modified Eagles Medium, poly-L-lysine, histamine and digitonin were from Sigma/Aldrich. Fetal bovine serum, and L-glutamine were from Life Technologies Corporation (Carlsbad, CA). TurboFect transfection reagent was from Fermentas (Thermo Fisher Scientific).

### Synthesis of CholPC and C6-Cer

CholPC and [^3^H]CholPC were prepared according to previously published methods [33]–[36]. The structure of CholPC was verified by ^1^H-NMR, and by by ESI-MS (MeOH): 574.45 [M+Na]^+^ [33]. C6-Cer or C6-[^3^H]Cer were prepared from hexanoic acid and sphingosine or [^3^H]sphingosine using *N,N'*-dicyclohexylcarbodiimide and triethylamine as catalysts [37]. Products were purified by preparative HPLC on a C18 phase, using methanol as solvent. Purity was assessed by analytical HPLC, and molecular identity by ESI-MS.

### Preparation of C6-Cer/CholPC or Chol/CholPC Bilayers

The synthesized CholPC and C6-Cer were kept as stock solutions in hexane-isopropanol (3∶2 by vol) and methanol, respectively, and stored at −20°C until used. Cholesterol was dissolved in hexane/isopropanol, and used without further purification. The mass of each compound was determined by weight prior to dilution in solvent. Bilayers of desired concentration were prepared from the stock solutions as follows: the appropriate amount of each lipid was put into a glass tube, dried under a flow of nitrogen at 40°C, redissolved in chloroform to ensure proper mixing and dried again. The dried lipid film was then hydrated in HBSS buffer (20 mM Hepes, 118 mM NaCl, 4.6 mM KCL, 1 mM CaCl_2_, 10 mM D-glucose, pH 7.4) at 55°C for 20 minutes and then sonicated for 10 minutes in a Branson 2510 bath sonicator (Branson Ultrasonics, Danbury, CT, USA) at the same temperature. The resulting dispersions of either C6-Cer/CholPC or Chol/CholPC (each prepared to a 1∶1 molar ratio) were kept at RT and used within 24 hours. For experiments with C6-Cer dissolved in DMSO, the dried lipid film was prepared as previously described, and dissolved in DMSO to give a 10 mM solution. As controls, cholesterol was also dissolved in DMSO and presented to cells, or was complexed with methyl-β-cyclodextrin (1∶8 molar ratio) as described previously [38].

### Cell Culture

Rat thyroid FRTL-5 cells, originally from the Interthyr Foundation (Bethesda, MD), were grown in Coon’s modified Ham’s F12 medium supplemented with 5% calf serum and six hormones (insulin, 10 µg/ml; transferrin, 5 µg/ml; hydrocortisone, 10 nM; tripeptide gly-L-his-L-lys, 10 ng/ml; TSH, 0.3 mU/ml; somatostatin, 10ng/ml) in a water-saturated atmosphere of 5% CO_2_ and 95% air at 37^o^C [39]. HeLa cells were grown in Dulbecco’s Modified Eagle Medium supplemented with fetal bovine serum (10%), L-glutamine (1%) and penicillin-streptomycin (1%) at 37°C and 5% CO_2_.

### Incorporation of Labeled [^3^H]CholPC and [^3^H]C6-Cer into FRTL-5 Cells

Near-confluent FRTL-5 cells were exposed for the indicated times to C6-Cer/CholPC bilayers in which either C6-Cer or CholPC was [^3^H]-labeled for indicated times. Alternatively, [^3^H]C6-Cer was presented to cells dissolved in DMSO. The final C6-Cer concentration was 0.05 mM. Zero time labeling was determined from cells exposed to labeled lipid for about 60 sec before rinsing and washing, and was subtracted from the 4 h labeling values for each lipid class. Dishes were rinsed with buffer 3 × 1 ml before freezing. Lipids from the cells were extracted with 1 ml hexane/isopropanol (3∶2 by vol) per dish for 20 min on a plate rocker. The lipid extract was transferred to a tube and dried under a flow of nitrogen at 40°C. The procedure was repeated one time to ensure efficient extraction of the lipids – extracts from each dish were pooled.

For analysis, the dried lipids were dissolved in 100 µl of hexane/isopropanol and 30 µl was applied onto a tlc plate (TLC PE SIL G Flexiplate; Whatman Ltd, Maidstone, Kent, England) using a Camag Automatic TLC Sampler III (Camag, Muttenz, Switzerland). Standard lipids (cholesterol, CholPC and cholesteryl oleate) were coeluted on the plates together with the samples. For CholPC, cholesterol and cholesterol oleate analysis, tlc plates were first eluted halfway with chloroform:methanol:acetic acid:water (5∶3:0.3∶0.3 by vol). The plates were dried, and eluted full length a second time with hexane, diethyl ether and acetic acid (13∶3:0.2 by vol). The plates were dried and the spots visualized using iodine vapor. The spots corresponding to CholPC, cholesterol, and cholesteryl oleate were cut and placed in scintillation tubes together with 3 ml of scintillation fluid (Optiphase 3, PerkinElmer/Wallac Turku, Finland) and left over night. The next day the radioactivity was counted using a LKB Wallac 1216 Rackbeta liquid scintillation counter (Wallac Oy, Turku, Finland).

### [3H]Thymidine Incorporation Assay

Cell proliferation was measured with [^3^H]-thymidine (Amersham, Buckinhamshire, UK) incorporation. Cells were plated on 35-mm plates (100 000 cells/plate) and grown for the times indicated together with 0.05 mM of C6-Cer (either as complex with CholPC or dissolved in DMSO). For the last 4 h, 0.4 µCi/ml [^3^H]-thymidine was added. The assay was stopped by washing the cells three times with ice-cold PBS. DNA was precipitated with ice-cold 5% *trichloroacetic* acid. The precipitate was dissolved in 0.1 M NaOH, and mixed with Optiphase Hisafe 3 scintillation liquid (PerkinElmer/Wallac Turku, Finland), and counted for activity.

### Cell Counting Assay

Cell proliferation was also measured by cell counting. Cells were plated on 35-mm plates (100 000 cells/plate) and grown for the indicated times with 0.05 mM of C6-Cer (either as a complex with CholPC or dissolved in DMSO). In control plates, only DMSO was used. At the indicated time, the cells were harvested using PBS containing 0.02% EDTA and 0.1% trypsin, and the cells were counted using trypan blue and a haemocytometer chamber.

### Cytosolic Calcium Measurements

HeLa cells were grown in 35-mm cell culture dishes containing 25-mm poly-L-lysine coated coverslips. At 50% confluence, the cells were exposed for 0.05 mM C6-Cer (either as complex with CholPC, or dissolved in DMSO) for 90 min. Then, the cells were washed three times with HBSS and incubated in HBSS with 2 µM Fura 2-AM for 30 min at room temperature. Extracellular Fura 2-AM was then removed by two washes followed by a 15 min incubation in HBSS. The coverslips were transferred to a heated chamber (37°C) and placed under an inverted Zeiss Axiovert 35 microscope equipped with a 40× Fluor objective and a SensiCam 12 bit CCD camera (PCO/CD Imaging, Kelheim, Germany). The source for excitation light was a XBO 75W/2 xenon lamp. Correct excitation wavelengths were produced using 340 and 380 nm filters which were controlled with a Lambda 10-2 device (Sutter Instruments, Novato, CA). Data was recorded using Axon Imaging Workbench 6.0 (Axon Instruments, Foster City, CA). For the treatments in Ca^2+^ free HBBS, CaCl_2_ was replaced with 0.150 mM EGTA.

### Mitochondrial Calcium Measurements

Recombinant aequorin targeted to the mitochondrial matrix (mtAEQ) and a purpose-built luminometer system were used for measurements of mitochondrial Ca^2+^ concentrations as described previously [40], [41]. The mtAEQ plasmid was a generous gift from professor Urs Ruegg (Geneva-Lausanne School of Pharmaceutical Sciences, University of Geneva, Switzerland). HeLa cells were grown on 13-mm poly-L-lysine coated coverslips and transfected with mtAEQ at 70% confluence using TurboFect transfection reagent. 24 hours after transfection the cells were preincubated for 180 min with 0.05 mM C6-Cer (either as complex with CholPC, or dissolved in DMSO). Cells were washed 3 times in HBSS and incubated for 1 h with 5 µM native colenterazine at room temperature. The coverslips were then placed into a heated (37°C) perfusion chamber and luminescence was measured. Data was recorded using EM6 Counter/Timer Software 2.5 (Electron Tubes Limited). After each experiment the cells were treated with 100 µM digitonin in HBSS containing 10 mM CaCl_2_ to obtain a maximal luminescence signal of the sample for calculating the Ca^2+^ concentration at each time point of the experiment as described in [40].

### Fluorescence-activated Cell Sorting for Apoptosis Detection

100,000 cells were grown on 35-mm plates and the media was collected after 48 h of incubation with ceramides (0.05 mM) and controls. FRTL-5 cells were detached with EDTA-trypsin solution and centrifuged along with the collected media. The pellet was incubated at room temperature for 15 min with 200 µl propidium iodide solution, containing 0.05 mg/ml propidium iodide, 3.8 µM sodium citrate, 0.1% Triton X-100 in PBS, at room temperature. The samples were then analyzed by flow cytometry using FACSCalibur and CellQuest Pro software (BD Biosciences, San Jose, CA, USA). The percentage of apoptotic cells was calculated using Flowing Software v 2.5 (www.flowingsoftware.com).

### Statistics

Each experiment was repeated at least three times. The results are expressed as the mean ± SEM. Statistical analysis was made using Student’s *t* test. When three or more means were tested, one-way ANOVA and Bonferronis’ *post hoc* test was used. A *P* value below 0.05 was considered as statistically significant.

## References

[1] Barenholz Y, Thompson TE (1980) Sphingomyelins in bilayers and biological membranes. Biochimica et Biophysica Acta 604: 129–158. 7000188 10.1016/0005-2736(80)90572-6

[2] Gault CR, Obeid LM, Hannun YA (2010) An overview of sphingolipid metabolism: from synthesis to breakdown. Adv Exp Med Biol 688: 1–23. 20919643 10.1007/978-1-4419-6741-1_1 PMC3069696

[3] Hannun YA, Luberto C, Argraves KM (2001) Enzymes of sphingolipid metabolism: from modular to integrative signaling. Biochemistry 40: 4893–903. 11305904 10.1021/bi002836k

[4] Ohvo-Rekila H, Ramstedt B, Leppimaki P, Slotte JP (2002) Cholesterol interactions with phospholipids in membranes. Prog Lipid Res 41: 66–97. 11694269 10.1016/s0163-7827(01)00020-0

[5] Hannun YA, Obeid LM (2011) Many ceramides. J Biol Chem 286: 27855–27862. 21693702 10.1074/jbc.R111.254359 PMC3151029

[6] Michel C, van Echten-Deckert G (1997) Conversion of dihydroceramide to ceramide occurs at the cytosolic face of the endoplasmic reticulum. FEBS Lett 416: 153–155. 9369202 10.1016/s0014-5793(97)01187-3

[7] Bartke N, Hannun YA (2009) Bioactive sphingolipids: metabolism and function. J Lipid Res 50 Suppl: S91–S96 10.1194/jlr.R800080-JLR200 PMC2674734 19017611

[8] Hassler DF, Bell RM (1993) Ceramidases: enzymology and metabolic roles. Adv Lipid Res 26: 49–57. 8379459

[9] Spiegel S, Cuvillier O, Edsall L, Kohama T, Menzeleev R, Olivera A, et al (1998) Roles of sphingosine-1-phosphate in cell growth, differentiation, and death. Biochemistry (Mosc ) 63: 69–73. 9526097

[10] Slotte JP (1997) Cholesterol-sphingomyelin interactions in cells–effects on lipid metabolism. Subcell Biochem 28: 277–93. 9090298

[11] Slotte JP (1999) Sphingomyelin-cholesterol interactions in biological and model membranes. Chem Phys Lipids 102: 13–27. 11001557 10.1016/s0009-3084(99)00071-7

[12] Westerlund B, Slotte JP (2009) How the molecular features of glycosphingolipids affect domain formation in fluid membranes. Biochim Biophys Acta 1788: 194–201. 19073136 10.1016/j.bbamem.2008.11.010

[13] Gangoiti P, Granado MH, Wang SW, Kong JY, Steinbrecher UP, et al (2008) Ceramide 1-phosphate stimulates macrophage proliferation through activation of the PI3-kinase/PKB, JNK and ERK1/2 pathways. Cell Signal 20: 726–736. 18234473 10.1016/j.cellsig.2007.12.008

[14] Gomez-Munoz A (2004) Ceramide-1-phosphate: a novel regulator of cell activation. FEBS Lett 562: 5–10. 15069950 10.1016/s0014-5793(04)00211-x

[15] Gomez-Munoz A (2006) Ceramide 1-phosphate/ceramide, a switch between life and death. Biochim Biophys Acta 1758: 2049–2056. 16808893 10.1016/j.bbamem.2006.05.011

[16] Adam D, Heinrich M, Kabelitz D, Schutze S (2002) Ceramide: does it matter for T cells? Trends Immunol 23: 1–4. 11801441 10.1016/s1471-4906(01)02091-9

[17] Okazaki T, Bell RM, Hannun YA (1989) Sphingomyelin turnover induced by vitamin D3 in HL-60 cells. Role in cell differentiation. J Biol Chem 264: 19076–19080. 2808413

[18] Zhang J, Alter N, Reed JC, Borner C, Obeid LM, et al (1996) Bcl-2 interrupts the ceramide-mediated pathway of cell death. Proc Natl Acad Sci U S A 93: 5325–5328. 8643573 10.1073/pnas.93.11.5325 PMC39244

[19] Hetz CA, Hunn M, Rojas P, Torres V, Leyton L, et al (2002) Caspase-dependent initiation of apoptosis and necrosis by the Fas receptor in lymphoid cells: onset of necrosis is associated with delayed ceramide increase. J Cell Sci 115: 4671–4683. 12415011 10.1242/jcs.00153

[20] Obeid LM, Linardic CM, Karolak LA, Hannun YA (1993) Programmed cell death induced by ceramide. Science 259: 1769–1771. 8456305 10.1126/science.8456305

[21] Venable ME, Lee JY, Smyth MJ, Bielawska A, Obeid LM (1995) Role of ceramide in cellular senescence. J Biol Chem 270: 30701–30708. 8530509 10.1074/jbc.270.51.30701

[22] Chalfant CE, Szulc Z, Roddy P, Bielawska A, Hannun YA (2004) The structural requirements for ceramide activation of serine-threonine protein phosphatases. J Lipid Res 45: 496–506. 14657198 10.1194/jlr.M300347-JLR200

[23] Chapman H, Ramstrom C, Korhonen L, Laine M, Wann KT, et al (2005) Downregulation of the HERG (KCNH2) K(+) channel by ceramide: evidence for ubiquitin-mediated lysosomal degradation. J Cell Sci 118: 5325–5334. 16263765 10.1242/jcs.02635

[24] Ramstrom C, Chapman H, Ekokoski E, Tuominen RK, Pasternack M, et al (2004) Tumor necrosis factor alpha and ceramide depolarise the resting membrane potential of thyroid FRTL-5 cells via a protein kinase Czeta-dependent regulation of K+ channels. Cell Signal 16: 1417–1424. 15381257 10.1016/j.cellsig.2004.05.007

[25] Perera MN, Ganesan V, Siskind LJ, Szulc ZM, Bielawski J, et al (2012) Ceramide channels: Influence of molecular structure on channel formation in membranes. Biochim Biophys Acta 1818: 1291–1301. 22365970 10.1016/j.bbamem.2012.02.010 PMC3319251

[26] Siskind LJ, Colombini M (2000) The lipids C2- and C16-ceramide form large stable channels. Implications for apoptosis. J Biol Chem 275: 38640–38644. 11027675 10.1074/jbc.C000587200 PMC2094390

[27] Siskind LJ, Kolesnick RN, Colombini M (2002) Ceramide channels increase the permeability of the mitochondrial outer membrane to small proteins. J Biol Chem 277: 26796–26803. 12006562 10.1074/jbc.M200754200 PMC2246046

[28] Kolesnick R, Hannun YA (1999) Ceramide and apoptosis. Trends Biochem Sci 24: 224–225. 10366847 10.1016/s0968-0004(99)01408-5

[29] Kolesnick R, Golde DW (1994) The sphingomyelin pathway in tumor necrosis factor and interleukin-1 signaling. Cell 77: 325–8. 8181053 10.1016/0092-8674(94)90147-3

[30] Singh D, Jarrell HC, Florio E, Fenske DB, Grant CW (1992) Effects of fatty acid alpha-hydroxylation on glycosphingolipid properties in phosphatidylcholine bilayers. Biochim Biophys Acta 1103: 268–274. 1543712 10.1016/0005-2736(92)90096-5

[31] Shabbits JA, Mayer LD (2003) Intracellular delivery of ceramide lipids via liposomes enhances apoptosis in vitro. Biochim Biophys Acta 1612: 98–106. 12729935 10.1016/s0005-2736(03)00108-1

[32] Stover T, Kester M (2003) Liposomal delivery enhances short-chain ceramide-induced apoptosis of breast cancer cells. J Pharmacol Exp Ther 307: 468–475. 12975495 10.1124/jpet.103.054056

[33] Lonnfors M, Langvik O, Bjorkbom A, Slotte JP (2013) Cholesteryl Phosphocholine - A Study on Its Interactions with Ceramides and Other Membrane Lipids. Langmuir. 10.1021/la3051324 [doi]. 10.1021/la3051324 23356741

[34] Gotoh M, Ribeiro N, Michels B, Elhabiri M, Albrecht-Gary AM, et al (2006) A novel type of membrane based on cholesteryl phosphocholine, cholesteryl phosphate, or sitosteryl phosphate, and dimyristoylglycerol. Chem Biodivers 3: 198–209. 17193258 10.1002/cbdv.200690023

[35] Bhatia SK, Hajdu J (1988) Stereospecifi synthesis of ether and thioether phospholipids. The use of L-glyceric acid as a chiral phospholipid precursor. J Org Chem 53: 5034–5039.

[36] Roodsari FS, Wu D, Pum GS, Hajdu J (1999) a new approach to the stereospecific synthesis of phospholipids. The use of L-glycerid acid for the preparation of diacylglycerols, phosphatidylcholines, and related derivatives. J Org Chem 64: 7727–7737.

[37] Nybond S, Bjorkqvist YJ, Ramstedt B, Slotte JP (2005) Acyl chain length affects ceramide action on sterol/sphingomyelin-rich domains. Biochim Biophys Acta 1718: 61–66. 16321609 10.1016/j.bbamem.2005.10.009

[38] Leppimaki P, Mattinen J, Slotte JP (2000) Sterol-induced upregulation of phosphatidylcholine synthesis in cultured fibroblasts is affected by the double-bond position in the sterol tetracyclic ring structure. Eur J Biochem 267: 6385–6394. 11029581 10.1046/j.1432-1327.2000.01726.x

[39] Ambesi-Impiombato FS, Parks LA, Coon HG (1980) Culture of hormone-dependent functional epithelial cells from rat thyroids. Proc Natl Acad Sci U S A 77: 3455–3459. 6106191 10.1073/pnas.77.6.3455 PMC349635

[40] Brini M, Marsault R, Bastianutto C, Alvarez J, Pozzan T (1995) etal (1995) Transfected aequorin in the measurement of cytosolic Ca2+ concentration ([Ca2+]c). A critical evaluation. J Biol Chem 270: 9896–9903. 7730373 10.1074/jbc.270.17.9896

[41] Brini M (2008) Calcium-sensitive photoproteins. Methods 46: 160–166. 18848993 10.1016/j.ymeth.2008.09.011

[42] Chapman JV, Gouaze-Andersson V, Messner MC, Flowers M, Karimi R, et al (2010) Metabolism of short-chain ceramide by human cancer cells–implications for therapeutic approaches. Biochem Pharmacol 80: 308–315. 20385104 10.1016/j.bcp.2010.04.001 PMC2883648

[43] Lange Y, Steck TL (2008) Cholesterol homeostasis and the escape tendency (activity) of plasma membrane cholesterol. Prog Lipid Res 47: 319–332. 18423408 10.1016/j.plipres.2008.03.001 PMC2659507

[44] Ramstrom C, Chapman H, Ekokoski E, Tuominen RK, Pasternack M, et al (2004) Tumor necrosis factor alpha and ceramide depolarise the resting membrane potential of thyroid FRTL-5 cells via a protein kinase Czeta-dependent regulation of K+ channels. Cell Signal 16: 1417–1424. 15381257 10.1016/j.cellsig.2004.05.007

[45] Satoh Y, Li X, Yokota H, Osada M, Ozaki Y, Katoh R, et al (2009) Regulation by sphingolipids of the fate of FRTL-5 cells. J Biochem 145: 31–36. 18953023 10.1093/jb/mvn138

[46] Tornquist K, Malm AM, Pasternack M, Kronqvist R, Bjorklund S, et al (1999) Tumor necrosis factor-alpha, sphingomyelinase, and ceramide inhibit store-operated calcium entry in thyroid FRTL-5 cells. J Biol Chem 274: 9370–9377. 10092616 10.1074/jbc.274.14.9370

[47] Rowland AA, Voeltz GK (2012) Endoplasmic reticulum-mitochondria contacts: function of the junction. Nat Rev Mol Cell Biol 13: 607–625. 22992592 10.1038/nrm3440 PMC5111635

[48] Wang G, Silva J, Krishnamurthy K, Tran E, Condie BG, et al (2005) Direct binding to ceramide activates protein kinase Czeta before the formation of a pro-apoptotic complex with PAR-4 in differentiating stem cells. J Biol Chem 280: 26415–26424. 15901738 10.1074/jbc.M501492200

[49] Blazquez C, Galve-Roperh I, Guzman M (2000) De novo-synthesized ceramide signals apoptosis in astrocytes via extracellular signal-regulated kinase. Faseb J 14: 2315–2322. 11053253 10.1096/fj.00-0122com

[50] Ruvolo PP (2003) Intracellular signal transduction pathways activated by ceramide and its metabolites. Pharmacol Res 47: 383–392. 12676512 10.1016/s1043-6618(03)00050-1

[51] Gulbins E, Dreschers S, Wilker B, Grassme H (2004) Ceramide, membrane rafts and infections. J Mol Med 82: 357–363. 15069600 10.1007/s00109-004-0539-y

[52] Gulbins E, Kolesnick R (2003) Raft ceramide in molecular medicine. Oncogene 22: 7070–7077. 14557812 10.1038/sj.onc.1207146

[53] Ganesan V, Colombini M (2010) Regulation of ceramide channels by Bcl-2 family proteins. FEBS Lett 584: 2128–2134. 20159016 10.1016/j.febslet.2010.02.032

[54] Colombini M (2010) Ceramide channels and their role in mitochondria-mediated apoptosis. Biochim Biophys Acta 1797: 1239–1244. 20100454 10.1016/j.bbabio.2010.01.021

[55] Shapiro D, Flowers HM (1962) Studies on Sphingolipids. VII. Synthesis and Configuration of Natural Sphingomyelins. J Am Chem Soc 84: 1047–1050.

[56] Lepple-Wienhues A, Belka C, Laun T, Jekle A, Walter B, et al (1999) Stimulation of CD95 (Fas) blocks T lymphocyte calcium channels through sphingomyelinase and sphingolipids. Proc Natl Acad Sci U S A 96: 13795–13800. 10570152 10.1073/pnas.96.24.13795 PMC24144

[57] Podbielska M, Krotkiewski H, Hogan EL (2012) Signaling and regulatory functions of bioactive sphingolipids as therapeutic targets in multiple sclerosis. Neurochem Res 37: 1154–1169. 22451227 10.1007/s11064-012-0728-y

[58] Stiban J, Fistere D, Colombini M (2006) Dihydroceramide hinders ceramide channel formation: Implications on apoptosis. Apoptosis 11: 773–780. 16532372 10.1007/s10495-006-5882-8

[59] Rodriguez-Acebes S, de la Cueva P, Fernandez-Hernando C, Ferruelo AJ, Lasuncion MA, et al (2009) Desmosterol can replace cholesterol in sustaining cell proliferation and regulating the SREBP pathway in a sterol-Delta24-reductase-deficient cell line. Biochem J 420: 305–315. 19260826 10.1042/BJ20081909 PMC2931812

[60] Slotte JP, Lundberg B (1983) Effects of cholesterol surface transfer on cholesterol and phosphatidylcholine synthesis in cultured rat arterial smooth muscle cells. Med Biol 61: 223–227. 6686272

[61] Detre C, Kiss E, Varga Z, Ludanyi K, Paszty K, et al (2006) Death or survival: membrane ceramide controls the fate and activation of antigen-specific T-cells depending on signal strength and duration. Cell Signal 18: 294–306. 16099142 10.1016/j.cellsig.2005.05.012

[62] Pinton P, Ferrari D, Rapizzi E, Di VF, Pozzan T, et al (2001) The Ca2+ concentration of the endoplasmic reticulum is a key determinant of ceramide-induced apoptosis: significance for the molecular mechanism of Bcl-2 action. EMBO J 20: 2690–2701. 11387204 10.1093/emboj/20.11.2690 PMC125256

[63] Roy SS, Madesh M, Davies E, Antonsson B, Danial N, et al (2009) Bad targets the permeability transition pore independent of Bax or Bak to switch between Ca2+-dependent cell survival and death. Mol Cell 33: 377–388. 19217411 10.1016/j.molcel.2009.01.018 PMC2662194

[64] Summers SA, Garza LA, Zhou H, Birnbaum MJ (1998) Regulation of insulin-stimulated glucose transporter GLUT4 translocation and Akt kinase activity by ceramide. Mol Cell Biol 18: 5457–5464. 9710629 10.1128/mcb.18.9.5457 PMC109130

[65] Zhou H, Summers SA, Birnbaum MJ, Pittman RN (1998) Inhibition of Akt kinase by cell-permeable ceramide and its implications for ceramide-induced apoptosis. J Biol Chem 273: 16568–16575. 9632728 10.1074/jbc.273.26.16568

[66] Siskind LJ, Davoody A, Lewin N, Marshall S, Colombini M (2003) Enlargement and contracture of C2-ceramide channels. Biophys J 85: 1560–1575. 12944273 10.1016/S0006-3495(03)74588-3 PMC1303332

[67] Yoshimura S, Banno Y, Nakashima S, Takenaka K, Sakai H, et al (1998) Ceramide formation leads to caspase-3 activation during hypoxic PC12 cell death. Inhibitory effects of Bcl-2 on ceramide formation and caspase-3 activation. J Biol Chem 273: 6921–6927. 9506997 10.1074/jbc.273.12.6921

[68] Koivusalo M, Jansen M, Somerharju P, Ikonen E (2007) Endocytic trafficking of sphingomyelin depends on its acyl chain length. Mol Biol Cell 18: 5113–5123. 17942604 10.1091/mbc.E07-04-0330 PMC2096594

[69] Koivusalo M, Alvesalo J, Virtanen JA, Somerharju P (2004) Partitioning of Pyrene-Labeled Phospho- and Sphingolipids between Ordered and Disordered Bilayer Domains. Biophys J 86: 923–935. 14747328 10.1016/S0006-3495(04)74168-5 PMC1303940

